# Blood biochemical and gut microbiotic neural network models forecasting human biological age

**DOI:** 10.18632/aging.206360

**Published:** 2026-03-11

**Authors:** Anastasia A. Kobelyatskaya, Olga N. Tkacheva, Alexandra A. Melnitskaia, Anna K. Ilyushchenko, Lubov V. Machekhina, Irina D. Strazhesko, Alexey Moskalev

**Affiliations:** 1 Russian Clinical Research Center for Gerontology, Pirogov Russian National Research Medical University, Ministry of Healthcare of the Russian Federation, Moscow 129226, Russia; 2 Institute of Biology of Aging and Healthy Longevity Medicine with Preventive Medicine Clinic, Petrovsky Russian Research Centre of Surgery, Moscow, Russia

**Keywords:** biological age, blood biochemistry, gut microbiome, neural network

## Abstract

Biological age reflects the current state of the body, considering the aspects of lifestyle, environment, and hereditary component. Currently there is no universal formula for determining it, but there are markers that can be used to calculate it. This study aims to develop and compare two models for calculating biological age based on laboratory blood tests and composition of gut microbiota. The biochemical model of biological age uses 7 indicators and is gender-specific (general – cystatin-C, IGF-1, DHEAS, only for females – homocysteine, urea, glucose, zonulin, only for males – HbA1c, NT-proBNP, free testosterone, hs-CRP). The microbial model requires the input of percentages of 45 bacterial species as indicators of the gut microbiota. Both methods demonstrate high predictive accuracy (MAE ~ 6 years, R2 > 0.8) and the degree of agreement of assessments both with each other and with PhenoAge (correlation > 0.89). For enhanced interpretability of the models, we applied the SHAP explanation algorithm, which allowed us to evaluate the contribution of each predictor to the final assessment of the biological age.

## INTRODUCTION

Biological age reflects an organism’s functional state, determined by comparing an individual’s physiological parameters to reference data for their corresponding chronological age group, and is expressed in years. In a healthy individual, biological age is expected to align with chronological age. However, lifestyle factors, adverse environmental exposures, and genetic predisposition can cause significant divergence between these measures. The assessment of biological age can incorporate diverse criteria, including morphological characteristics, biochemical profiles of bodily fluids, and cardiovascular health metrics. The relative importance of these criteria varies across an individual’s lifespan [1]. To date, no singular method exists that accurately and reliably quantify the biological age of the entire organism or its specific systems [2]. Previously, methods based on DNA analysis were considered among the most accurate for predicting chronological age, achieving mean absolute errors of under three years. However, these approaches require sophisticated equipment and specialized personnel, limiting their widespread adoption in clinical practice. Recent research has clarified that such molecular methods represent just one component of a comprehensive biological age assessment [3]. Consequently, there is a pressing need to develop accurate methods for determining biological age using readily available patient data [4, 5]. Furthermore, a critical paradigm shift is required: both single biomarkers and composite panels must transition from population-based reference ranges towards truly personalized assessments [6].

Among the pioneering aging clocks were Horvath’s and Hannum’s clocks, which primarily leveraged epigenetic markers [7, 8]. Subsequently, more comprehensive approaches integrating clinical parameters emerged, such as Levine’s PhenoAge [9] and GrimAge [10]. While these solutions estimate general, organism-level biological age, other models focus on specific systems or functions. Examples include tools like Arterial Indices [11], AI ECG-heart age [12], and EchoAGE [3]. Currently, several established approaches utilize blood test parameters to assess biological age, such as PhenoAge and DunedinPACE [9, 13]. These models incorporate common clinical biomarkers—including albumin, creatinine, glucose, glycated hemoglobin, and white blood cell counts—to provide a holistic assessment of the organism. Other models are also based on blood tests target specific systems, such as metabolism [14] or immune status [15]. Additionally, methods for estimating biological age from gut microbiome data have been developed, exemplified by gAge and the Viome Aging Clock [16, 17]. This study aims to develop, interpret, and compare calculators of biological age based on blood biochemical parameters and taxonomic features of the gut microbiota.

## MATERIALS AND METHODS

### Cohorts

This work included pseudonymized laboratory blood and microbiome data obtained from 637 patients without age-associated diseases (Table 1).

**Table 1:** Characteristics of the studied cohort.

	**Male**	**Female**	**Whole**
Patients, n	152	485	637
Age, mean (range), years	47 (18-95)	56 (18-99)	54 (18-99)

### Receiving laboratory blood and microbiome data

Obtaining blood samples and performing biochemical tests. Venous blood was collected into various test tubes. All samples, except for those in whole blood tubes, were centrifuged for 10 minutes at 3,000 RPM. The analyzed parameters included adiponectin, markers of carbohydrate and lipid metabolism, cellular aging, endothelial dysfunction, extracellular matrix status, complete blood count (CBC), hormonal and vitamin status, immune aging, inflammation, integrity of cellular barriers, mitochondrial dysfunction, and standard serum biochemical parameters (Supplementary Table 1A).

Obtaining stool samples, library preparation, sequencing, and data processing. Stool samples were collected using Nobias Stool Collection tubes, which maintain the stability of the intestinal microbiome at room temperature for several weeks prior to laboratory transfer. DNA was extracted from the stool samples using DNA isolation kits (Nobias Technologies, Russia), which included a step for sample homogenization with solid-state microparticles and removal of inhibitors. The quantity of 16S rRNA gene copies in the isolated DNA was determined using quality control kits for metagenomic studies at the preanalytical stage (Nobias Technologies, Russia). The full-length 16S rRNA gene was amplified using primers 27F and 1492R (AGAGTTTGATYMTGGCTCAG and GGTTACCTTGTTAYGACTT, respectively) and a CFX 96 amplifier (Bio-Rad, USA). The resulting PCR products were purified using Agencourt AMPure XP magnetic beads (Beckman Coulter Inc., USA). The quality of the amplicons was assessed by electrophoresis on a 1.5% agarose gel. Subsequent amplicon library preparation and sequencing were performed using New England Biolabs (NEB) reagents: single-strand break and end repair with the “NEBNext FFPE Repair Mix” (M6630) and “NEBNext End Repair/dA-Tailing Module” (E7546), followed by adapter ligation using the “NEBNext Quick Ligation Module” (E6056). All enzymatic (intermediate) steps in library preparation included necessary sample purification steps using Agencourt AMPure XP magnetic beads (Beckman Coulter Inc.). The concentration of the final 16S rRNA libraries was measured with a Qubit fluorimeter (Invitrogen, USA) using the Quant-iT™ High-Sensitivity dsDNA Assay Kit (Thermo Fisher Scientific, USA). Purified libraries were pooled in equimolar ratios based on their quantified concentrations. Sequencing was carried out using Oxford Nanopore Technologies kits: “Ligation Sequencing Kit” (SQK-LSK109), “Flow Cell Priming Kit” (EXP-FLP002), and the “Native Barcoding Expansion 96” kit (EXP-NBD196) for PCR-free multiplexing, on a MinION device with an R9 flow cell (FLO-MIN106). Basecalling was performed using Guppy (version 5.1.13) in high-accuracy mode with a minimum quality cutoff of Q-score 7. Barcode removal and read quality assessment were conducted using PoreChop [18] and NanoFilt [19]. Reads shorter than 1400 bp and with a quality score below 10 were excluded from the analysis. To mitigate the impact of sequencing depth on the detection of low-abundance microbes, a rarefaction to 9,000 reads per sample was performed. Samples with insufficient sequencing depth were excluded from further analysis. Read mapping to the NCBI database was performed using the EMU software [20]. Abundance tables at the species, genus, and family levels were generated by summing the abundances of species belonging to the corresponding taxonomic group.

### Data analysis

Statistical analysis was performed in the R environment [21]. Power analysis was conducted using the pwr.r.test function (r = 0.3, sig.level = 0.05, power = 0.90, alternative = two.sided) from the pwr library. Spearman’s correlation analysis, as a non-parametric test, was employed to assess the strength of the relationship between variables and age. Correlation analysis was performed in several modes: for the entire sample, separately for the male group, and separately for the female group, using the cor.test function [22]. Prior to statistical analysis and model creation/training, the study sample was divided into training and test datasets in a 2:1 ratio. Input data from both the training and test datasets were scaled and centered. Multicollinearity was checked using the check_collinearity function from the performance package. Principal Component Analysis (PCA) and PERMANOVA were performed using the prcomp function (stats package) and the adonis2 function (vegan package), respectively [23]. Visualization of the results was carried out using the ggplot2 package [24].

Model Development. A Fully Connected Neural Network (FCNN) architecture was implemented as the model using the Keras [25] and TensorFlow [26] libraries. The model architecture is a deep network containing 10 hidden layers. The input layer consists of the variable values. Each hidden layer contained between 20 and 750 neurons with a swish activation function. The output layer was a single neuron with a linear activation function. Mean Squared Error (MSE) was used as the loss function. The Adam algorithm [27] with a learning rate of 0.003 was used as the optimizer. To prevent overfitting, the model was checkpointed after each epoch, and the number of epochs yielding the highest model quality was considered optimal (with a maximum of 500 epochs). The metrics for evaluating model quality during training were Mean Absolute Error (MAE), Mean Squared Error (MSE), Median Absolute Error (MedAE), Root Mean Squared Error (RMSE), Coefficient of Determination (R^2^), and ε-accuracy (where ε = 10, representing an accuracy spread of ±10 years). These metrics were calculated using the caret R package [28].

For post-processing of the developed models, Explainable Artificial Intelligence (XAI) methods were applied, specifically the SHapley Additive exPlanations (SHAP) algorithm, to interpret the model’s predictions using the kernelshap [29] and shapviz [30] libraries.

## RESULTS

### Biochemical parameters as potential predictors of biological age

**Figure 1:**
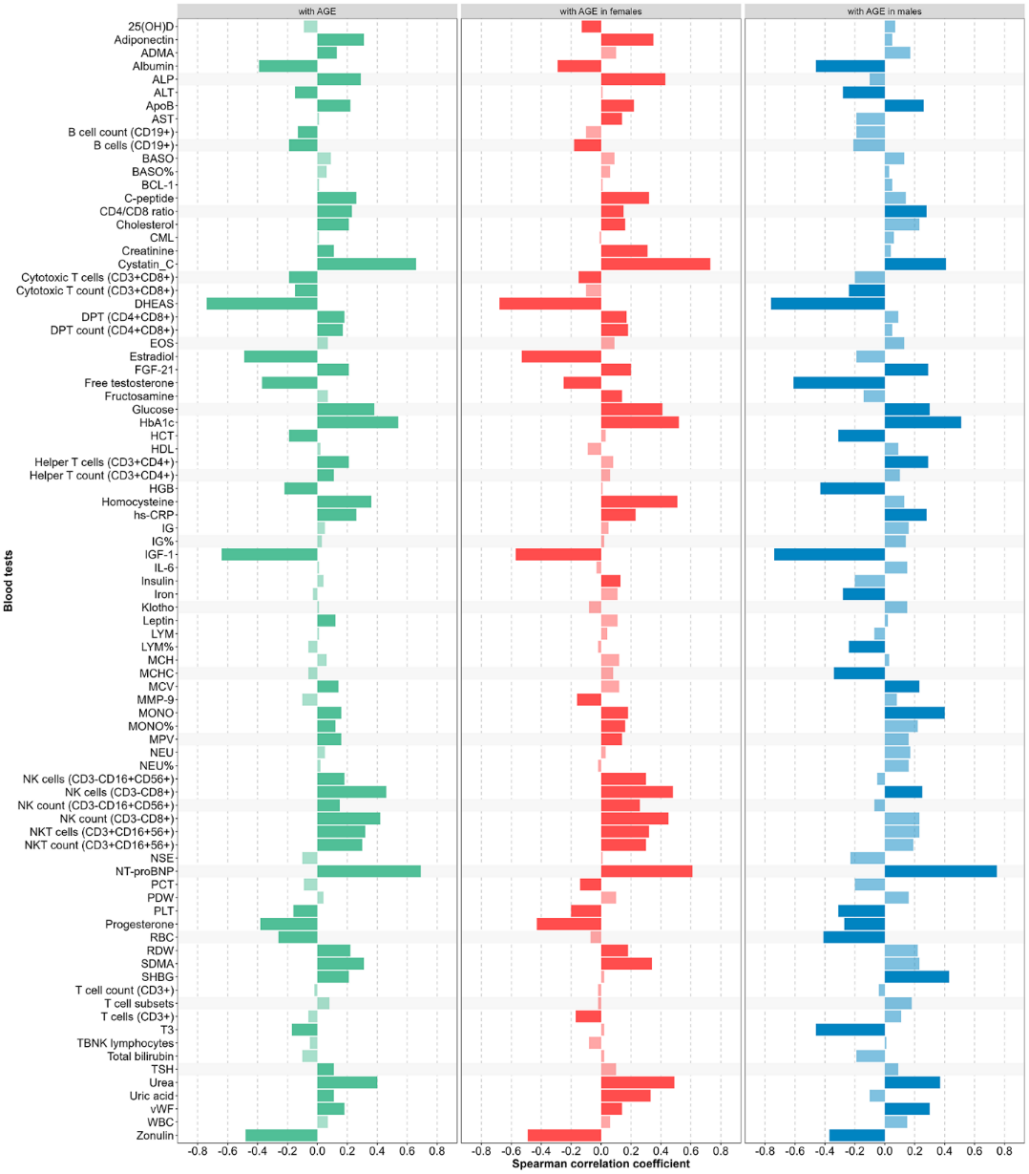
Correlation coefficient between blood tests and age (green – in training subset, red – in female, blue – in male, light green, red, blue colors – not significant).

To analyze the relationship between 85 blood parameters and age, a nonparametric correlation analysis was performed for the training subset (N=401), as well as for groups of men and women separately (Figure 1, and Supplementary Table 1B).

Based on the results of the correlation analysis, we selected the seven optimal predictors for each gender, giving preference to the most widespread and clinically accessible parameters, for females: homocysteine, cystatin-C, IGF-1, DHEAS, urea, glucose, zonulin, and for males: DHEAS, HbA1c, IGF-1, NT-proBNP, free testosterone, cystatin-C, hs-CRP.

**Figure 2:**
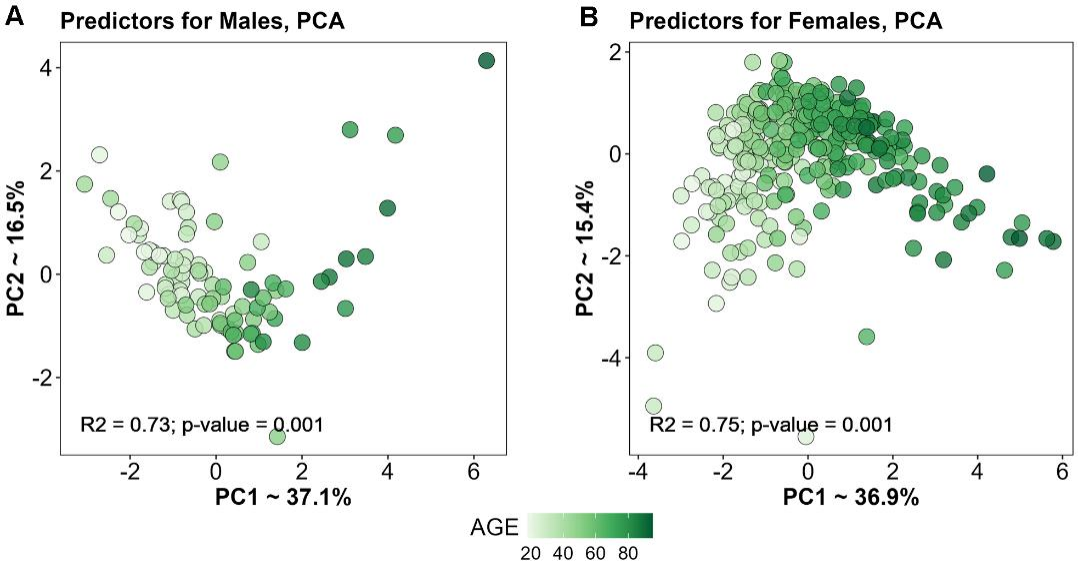
PCA based on selected predictors for Males (**A**) and Females (**B**).

We assessed multicollinearity for the selected predictors separately in men and women. As shown in Supplementary Table 1C, there was no substantial multicollinearity in either group. Using these predictors, we performed PCA and evaluated the association between age and the distribution in principal component space separately for men and women (Figure 2).

### Creating a predictive model based on blood parameters

**Figure 3:**
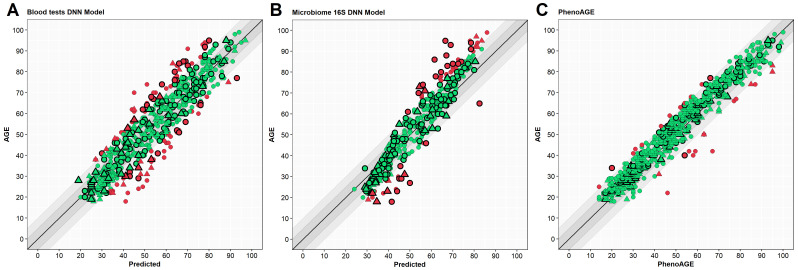
**Scatter plots of results from different models.** Y-axis – Age (years), X-axis – predicted age. Circle – women, triangle – men. Green – case in a 10-year spread, red – out of spread. Points with border – test set cases. (**A**) – blood tests model, (**B**) – microbiome model, (**C**) – PhenoAge. Outlined shapes – test subset samples.

To create the model, we used the values of the seven laboratory blood parameters listed above, which served as the input layer of the neural network. Since the previous stages of the analysis indicated the feasibility of a sex-specific mode, we trained two models for each sex. The final age-prediction model demonstrated high accuracy on both the training and test datasets (Table 2 and Figure 3A).

**Table 2:** Quality indicators of the final model designed to estimate biological age based on blood tests.

**Data set**	**MSE**	**MAE**	**MedAE**	**RMSE**	**rho**	**R2**	***ε*-acc10**
Both sexes							
Train	65.6	6.3	5.1	8.1	0.92	0.84	0.81
Test	62.2	6.4	5.5	7.9	0.92	0.84	0.80
Males							
Train	56.2	5.8	4.2	7.5	0.93	0.86	0.86
Test	52.0	6.1	6.1	7.2	0.92	0.85	0.82
Females							
Train	68.4	6.4	5.5	8.3	0.90	0.82	0.80
Test	65.0	6.4	5.0	8.1	0.91	0.83	0.80

Train, training set (N=401); Test, test set (N=156); MSE, Mean Square Error; MAE, Mean Absolute Error; MedAE, Median absolute error; RMSE, Root Mean Square Error; R2, Determination Coefficient; rho, Spearman correlation coefficient; *ε*-acc, *ε*-precision or epsilon precision, where *ε* = 10, i.e. ± 10 years is the spread when evaluating accuracy.

### Bacterial species as potential predictors

After the initial processing of 16S sequencing data, a matrix with a percentage of bacterial species was obtained. Since the bacterial representation in the biomaterial of the cohort was highly variable, only species present in at least 10% of the patients in the cohort were used for further analysis.

**Figure 4:**
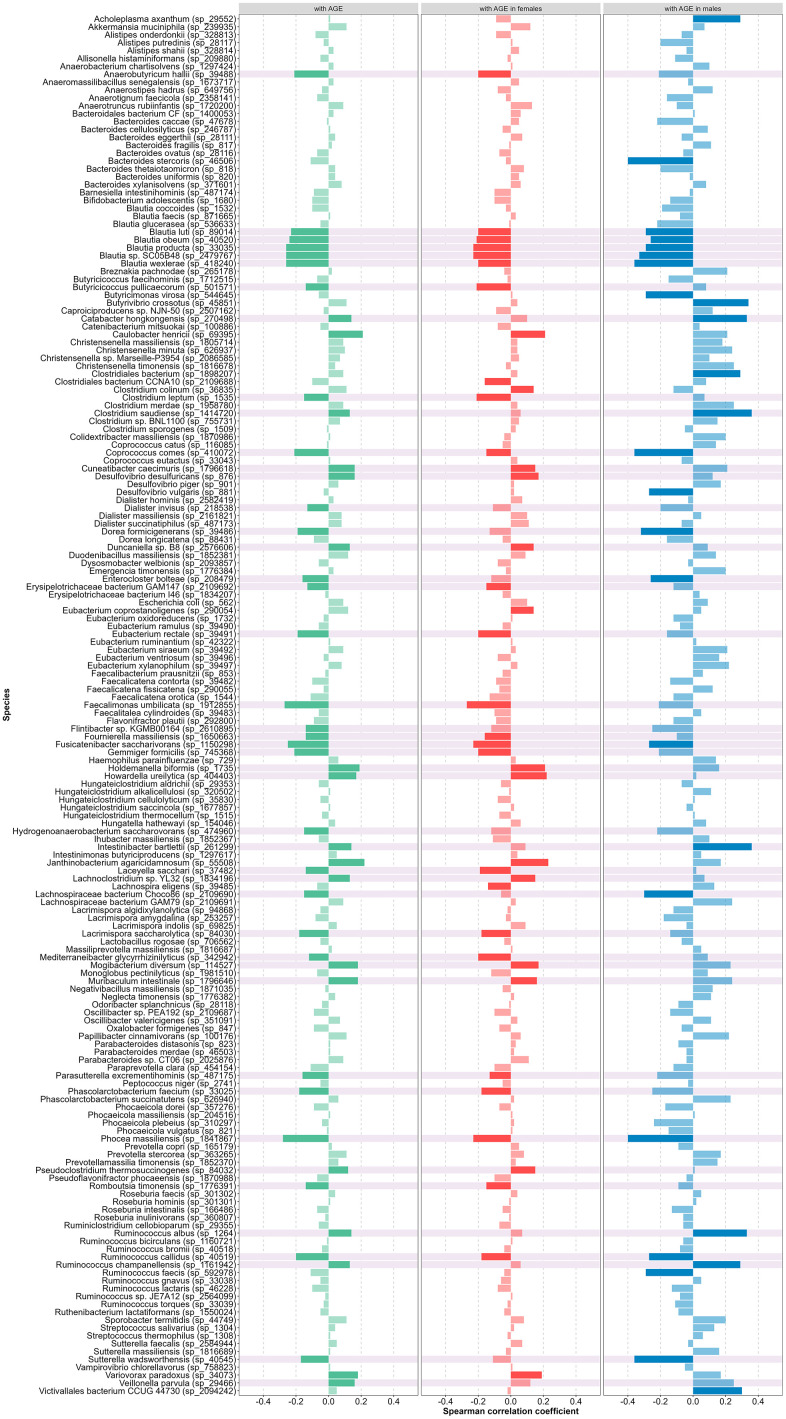
Correlation coefficient between species presence and age (green – in all cohort, red – in female, blue – in male, light green, red, blue colors – not significant, background color – selected as predictors).

To identify the relationship of individual representatives of the microbiota with age, a nonparametric correlation analysis was performed for the training subset (N=281), as well as for groups of men and women separately (Figure 4 and Supplementary Table 1D).

**Figure 5:**
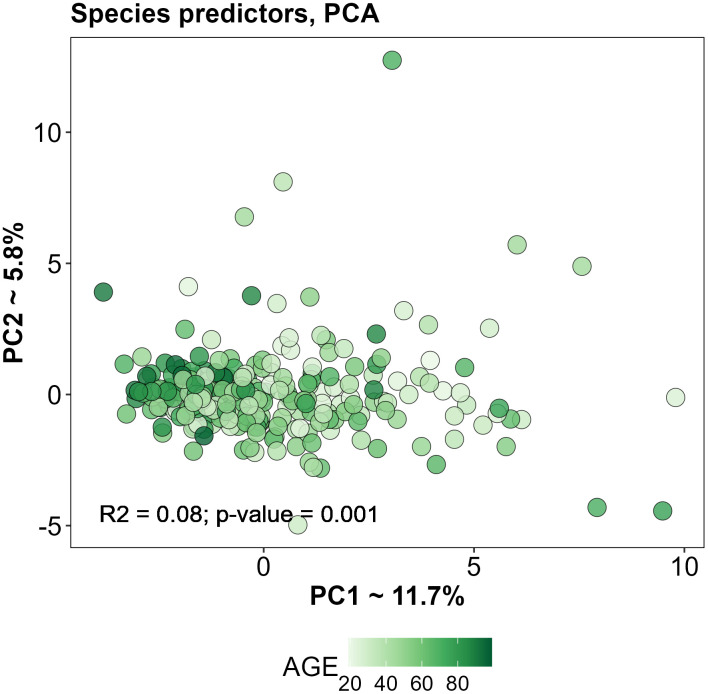
PCA based on selected predictors for training subset.

We assessed multicollinearity for the selected predictors separately in men and women. As shown in Supplementary Table 1E, there was no substantial multicollinearity in either group. Using these predictors, we performed PCA and evaluated the association between age and the distribution in principal component space separately for men and women (Figure 5). Overall, no significant sex differences in species abundance were detected. Therefore, based on the results of the whole cohort, we selected 45 species to build the model (they are highlighted in Figure 4).

### Creating a predictive model based on microbiota

To create the model, we used the relative abundances of the 45 selected bacterial species as indicators of the gut microbiota, which served as an input layer for the neural network. Unlike blood markers, microbiota indicators showed a less pronounced direct relationship with age. Therefore, the output of the microbiota model served as a normalized value relative to the average chronological age of the cohort, and this resultant value was defined as the “microbiotic age.” All subsequent calculations and analyses are based on this microbiotic age. The final age-prediction model demonstrated the accuracy shown in Table 3 (Figure 3B).

**Table 3:** Quality indicators of the final model designed to estimate biological age based on gut microbiota.

**Data set**	**MSE**	**MAE**	**MedAE**	**RMSE**	**rho**	**R2**	***ε*-acc10**
Both sexes							
Train	41.1	5.2	4.5	6.4	0.97	0.95	0.88
Test	86.1	7.1	5.5	9.3	0.91	0.84	0.78
Males							
Train	33.4	4.6	4.0	5.8	0.98	0.97	0.90
Test	65.4	6.4	5.5	8.1	0.93	0.86	0.83
Females							
Train	43.3	5.3	4.5	6.6	0.97	0.94	0.87
Test	93.1	7.3	5.5	9.7	0.90	0.81	0.77

Train, training set (N=281); Test, test set (N=141); MSE, Mean Square Error; MAE, Mean Absolute Error; MedAE, Median absolute error; RMSE, Root Mean Square Error; R2, Determination Coefficient; rho, Spearman correlation coefficient; *ε*-acc, *ε*-precision or epsilon precision, where *ε* = 10, i.e. ± 10 years is the spread when evaluating accuracy.

### Evaluation by PhenoAge

In addition to verification using our models, the cohort under study had the necessary data for evaluation by the popular PhenoAge method (formula in the article) [9]. According to the results described above, we also calculated statistics for these predictions (Table 4 and Figure 3C).

**Table 4:** Indicators of PhenoAge.

**Data set**	**MSE**	**MAE**	**MedAE**	**RMSE**	**rho**	**R2**	***ε*-acc10**
Whole	25.8	3.9	3.5	5.1	0.97	0.95	0.96
Males	16.1	3.2	2.5	3.9	0.98	0.96	0.98
Females	29.3	4.3	4.0	5.4	0.97	0.95	0.97

MSE, Mean Square Error; MAE, Mean Absolute Error; MedAE, Median absolute error; RMSE, Root Mean Square Error; R2, Determination Coefficient; rho, Spearman correlation coefficient; *ε*-acc, *ε*-precision or epsilon precision, where *ε* = 10, i.e. ± 10 years is the spread when evaluating accuracy.

### Comparison of all three models

**Figure 6:**
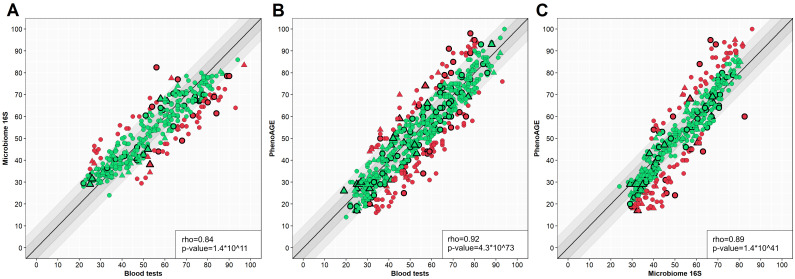
**Scatter plots of different model results comparison.** Y-axis – one model, X-axis – another model. Circle – women, triangle – men. Green – case in a 10-year spread, red – out of spread. Points with border – test set cases. (**A**) – blood tests model versus microbiome model, (**B**) – blood tests model versus PhenoAge, (**C**) – microbiome model versus PhenoAge. Outlined shapes – test subset samples. The correlation coefficients are presented for the test set.

As all three biological age assessment methods were applied to the same patients, we evaluated the consistency of their results (Supplementary Table 1F and Figure 6). All three models showed strong correlation with chronological age (r > 0.9). As seen in Figure 6, all three models demonstrated strong agreement with each other, with inter-model correlations reaching 0.84 (for test subset).

### Explanation of the final model’s operation

**Figure 7:**
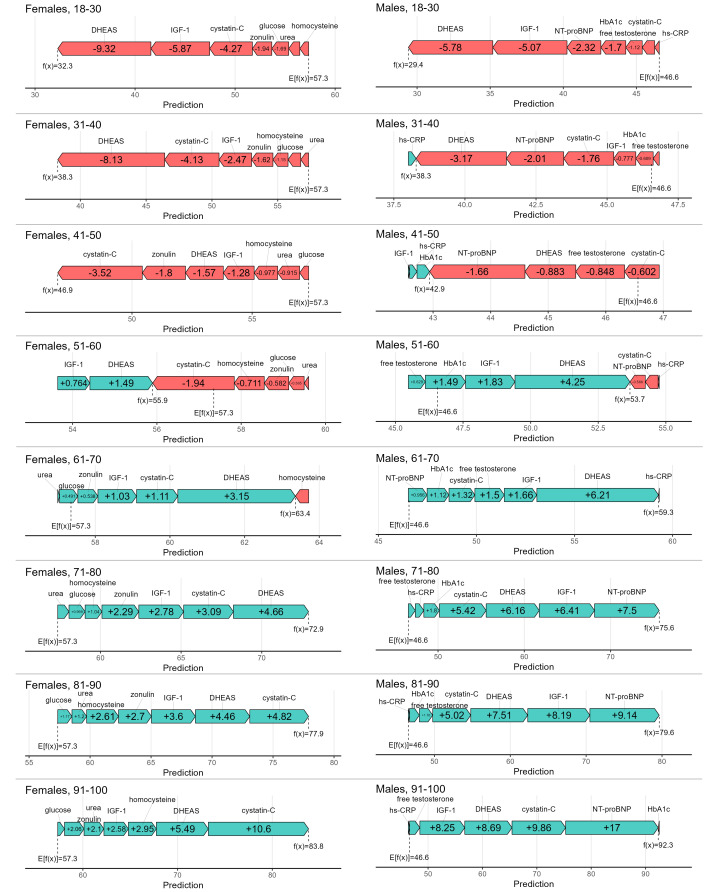
Collapsed SHAP values (in years) of each predictor in age groups for females and males.

**Figure 8:**
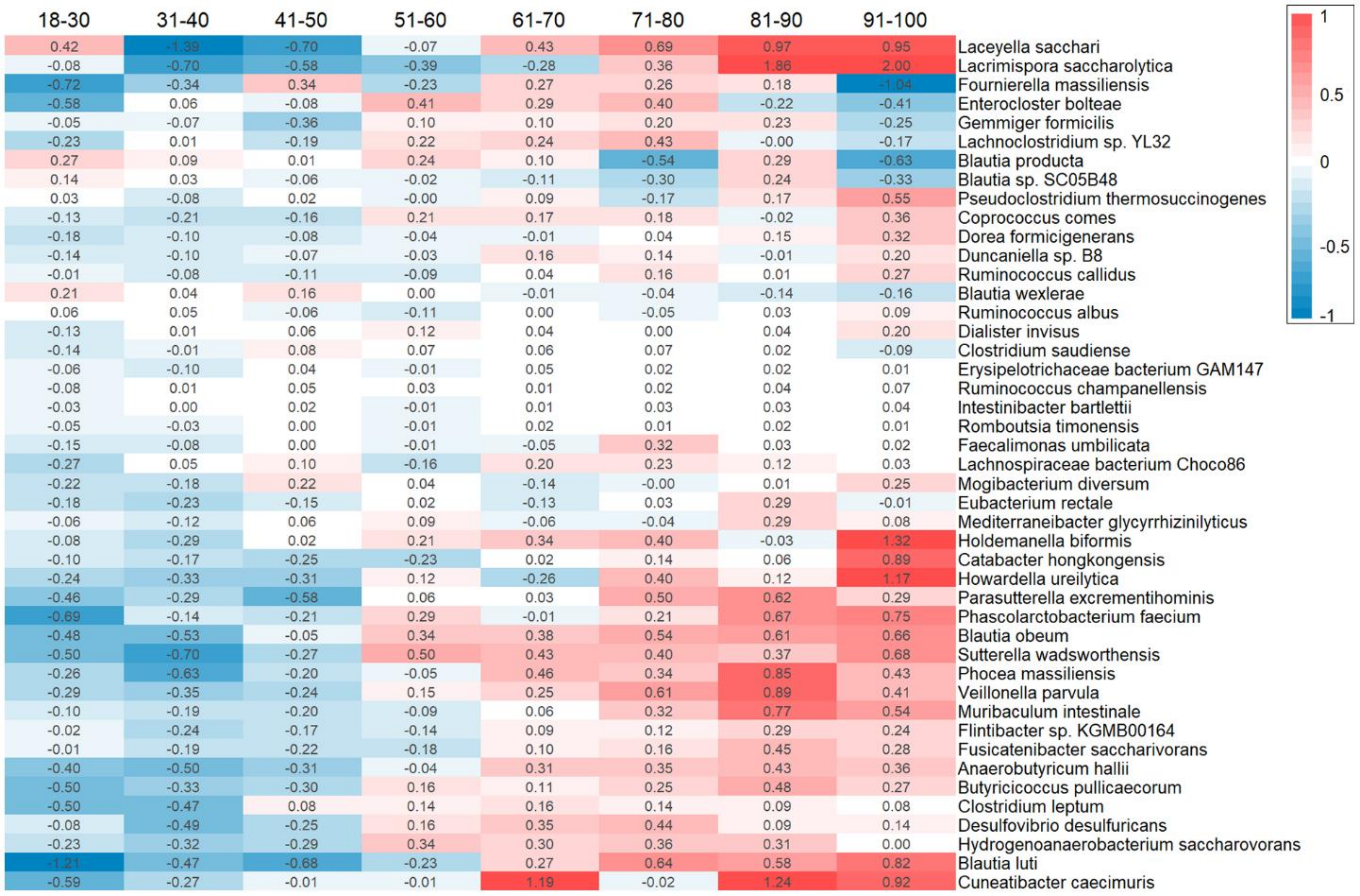
Heatmap with collapsed SHAP values (in years) of each species in age groups.

To gain detailed insight into the functioning of the biological age assessment models, we employed post-hoc Explainable Artificial Intelligence (XAI) technology, transforming the previously “black box” models into interpretable “white box” models. The SHapley Additive exPlanations (SHAP) approach was used to determine the contribution of each predictive variable to the overall estimate by calculating SHAP values (expressed in years) for each individual case (Figures 7, 8). The algorithm uses the cohort’s average age as the baseline expectation: for the biochemical model, this was 57.3 years for women and 46.6 years for men in the training subset; for the microbiota model, the average age of the training subset was 54 years. The SHAP values of the predictors (positive or negative) are then added to this baseline value to arrive at the model’s predicted age.

Based on the SHAP values from the biochemical model, the DHEAS parameter had the most significant impact on predicting biological age in both sexes. In younger individuals, it typically decreases the predicted age, whereas in older individuals, it increases it. Other indicators influenced biological age estimates in a similar directional manner. However, for the oldest age group, cystatin C had the highest weight for women, and NT-proBNP for men.

Interpreting the contribution of bacterial species to the prediction of microbiotic age is more complex than for biochemical parameters. The model uses the relative abundances of 45 species, and none acts as a single dominant or core predictor. Most predictors influence the microbiotic age estimate within an average range of ±0.5 to 1.5 years. The predictors in Figure 8 are ranked by the gradient of their contribution across age groups. For instance, *Blautia obeum* and *Butyricicoccus pullicaecorum* show the most explicit gradient of change. Most species displayed exhibit a clear trend towards increasing the predicted age as their SHAP value changes. It is important to note that *Blautia obeum* and *Butyricicoccus pullicaecorum* are negatively correlated with age, meaning lower abundances of these species are associated with a higher predicted age. However, there are several species whose contributions do not change unidirectionally but rather show saltatory patterns for specific age groups, such as *Blautia* sp. and *Howardella ureilytica*.

## DISCUSSION

In this work, we focused on creating and comparing two models for determining biological age based on paired biomedical data from laboratory blood tests and gut microbiota indicators. To achieve this, we performed a correlation analysis and selected appropriate predictors. Consequently, for the biochemical model of biological age, we used 7 indicators: 3 common markers (cystatin C, IGF-1, DHEAS), alongside 4 specific to females (homocysteine, urea, glucose, zonulin) and 4 specific to males (HbA1c, NT-proBNP, free testosterone, hs-CRP). The microbiota-based model of biological age incorporated the relative abundances of 45 bacterial species as predictors. Both models demonstrated high accuracy (MAE ~ 6-7 years, R^2^ > 0.8). To enhance model interpretability, we applied Explainable Artificial Intelligence (XAI), specifically the SHAP explanation algorithm, which allowed us to evaluate the contribution of each predictor to the final biological age estimate.

Among the common indicators, cystatin C levels increase with age, while IGF-1 and DHEAS decrease. Cystatin C is an inhibitor of cysteine proteases and a marker of renal excretory function. Its elevated level indicates reduced glomerular filtration rate and, consequently, impaired kidney function, which carries cardiovascular risks [31]. IGF-1 (or somatomedin C) stimulates the growth of bones and soft tissues and is responsible for maintaining muscle mass. Its decline contributes to reduced muscle mass (sarcopenia) and the development of frailty [32]. DHEAS possesses neurosteroid activity, modulating the action of GABA and other receptors. Its decrease leads to an imbalance in the GABAergic system and excessive inhibition, slowing cognitive processes [33]. Furthermore, in women, levels of homocysteine, urea, and glucose increase, while zonulin levels decrease. Homocysteine is an intermediate product in the metabolism of the amino acids methionine and cysteine. Elevated blood homocysteine exerts pro-atherogenic and pro-thrombotic effects on the endothelium and is associated with an increased risk of Alzheimer’s disease and senile dementia [34]. Urea is the final product of protein breakdown, excreted by the kidneys, and serves as an indicator of renal excretory capacity. An increase in its level indicates declining kidney function. Glucose is the primary energy source for cells. High glucose levels are linked to the development of insulin resistance and diabetes, accompanied by an elevated risk of cardiovascular diseases. Zonulin controls intestinal barrier permeability by regulating the tightness of intercellular junctions. Increased zonulin levels lead to heightened permeability. However, lower zonulin levels are less studied; they may cause age-related physiological hypopermeability, potentially leading to impaired nutrient absorption and malabsorption, immune system dysfunction due to reduced stimulation (increasing the risk of allergies and autoimmune diseases), and depletion of the microbiome [35]. In men, levels of HbA1c, NT-proBNP, and hs-CRP increase, while free testosterone decreases. HbA1c reflects the average blood glucose level over several months and is a marker of chronic hyperglycemia. Its high value is a risk factor for age-associated diseases. NT-proBNP indicates the processing of BNP in response to cardiac wall stress, regulating blood volume through modulation of blood pressure. Its elevated level suggests myocardial strain and heart insufficiency. hs-CRP is an acute-phase protein produced by the liver in response to IL-6 and other pro-inflammatory cytokines, serving as a marker of chronic, low-grade inflammation (inflammaging) [36]. Free testosterone acts on androgen receptors, regulating anabolic processes and libido. Its age-related decline indicates andropause and is a factor in the development of sarcopenia and frailty. DHEAS, cystatin C, and NT-proBNP exerted the strongest influence on the prediction dynamics within the model. Overall, these interconnected processes reflect systemic aging of the metabolic, endocrine, and excretory systems, leading to sarcopenia, osteoporosis, insulin resistance, vascular damage, chronic inflammation, toxin accumulation, and, consequently, accelerated aging [37–39].

Among the selected 45 gut bacterial species, 16 were positively associated with age. Of these, 3 species (Muribaculum intestinale, Ruminococcus albus, Ruminococcus champanellensis) can be considered “beneficial,” as they are involved in acetate production, carbohydrate fermentation, and support overall microbiota and metabolic health. However, 5 other species (Catabacter hongkongensis, Clostridium saudiense, Desulfovibrio desulfuricans, Holdemanella biformis, Howardella ureilytica) are potentially pathogenic and may cause infections or contribute to inflammatory bowel disease involving an immune component. The remaining 8 positively associated species can be classified as neutral, as they produce acetate, butyrate, and propionate, and modulate metabolic pathways. The majority of microorganisms (29 species) exhibited a negative correlation with age, meaning their abundance decreases in older age. Among these, 7 species (Anaerobutyricum hallii, Butyricicoccus pullicaecorum, Clostridium leptum, Coprococcus comes, Eubacterium rectale, Fusicatenibacter saccharivorans, Lachnospiraceae bacterium Choco86) can be considered beneficial. They are responsible for synthesizing or fermenting various substances, support barrier function, exert anti-inflammatory effects, and reduce the risk of metabolic disorders. Conversely, only 5 species (Blautia obeum, Blautia producta, Dialister invisus, Enterocloster bolteae, Sutterella wadsworthensis) are potentially pathogenic, potentially contributing to obesity, IBS, and negatively impacting mental health. Most of the remaining age-negatively correlated species can be classified as neutral; they produce and ferment substances but under certain conditions may cause gastrointestinal disorders and metabolic disturbances. The bacterial species used in the model collectively reflect an age-related decline in protective and metabolic functions, an increase in pro-inflammatory potential, and a disruption and impoverishment of metabolic networks. The microbiome composition shifts towards a reduced abundance of beneficial bacteria and an increased abundance of potentially pathogenic ones, decreasing the production of butyrate and acetate, distorting metabolic functions, and elevating the risk of disease development [40].

Several other models incorporate the aforementioned biochemical markers. For instance, the widely known PhenoAge (cl004) also includes glucose and CRP among its 9 blood parameters. However, besides these 9 parameters, the PhenoAge formula includes chronological age as an input, which complicates determining the specific contribution of the biomarkers themselves to the calculated phenotypic age. The most frequently used markers in published formulas are glucose, HbA1c, hs-CRP, and urea. These are included in models such as AgeML (cl062) [41], Deep Longevity Aging Clock (cl068) [42], Elastic-Net Cox (ENC cl082) [43], among others, which incorporate subsets of these markers. The mentioned formulas utilize between 16 and 62 biochemical parameters, supplemented by physical parameters or age (as in PhenoAge). These models employ either regression or neural network approaches, were trained on narrower age ranges, and demonstrate R^2^ values ranging from 0.4 to 0.8. None of these approaches provide comprehensive global and local explanations for their predictions (XAI). A number of models based on microbiota indicators are known, including the Viome Aging Clock (cl069) [17], Human Gut Microbiome Aging Clock (cl103) [44], Ensemble model for gut microbiome aging clock (cl104) [45], and gAge (cl105) [16]. Some of these formulas additionally use gene expression levels or pathway enrichment data. Only the Ensemble model for gut microbiome aging clock (cl104) [45] also utilizes some of the bacterial species we identified (Clostridium leptum, Coprococcus comes, Dorea formicigenerans, Parasutterella excrementihominis, Sutterella wadsworthensis, Veillonella parvula). The aforementioned models are built using 100-1000 predictors, and their performance varies (MAE: 5.9–8.6 years, R^2^: 0.29–0.6). However, similar to the biochemical models, none of them explain the contribution of specific bacterial abundances to the biological age estimate for an individual patient. Both of our models were trained on the same cohort, and the high correlation between the estimates from the two models suggests shared underlying aging mechanisms. Potential interconnected “axes” include: the inflammaging axis - Characterized by elevated levels of hs-CRP (an inflammation marker) and an increased abundance of potentially pathogenic species that can provoke gut inflammation. The metabolic dysregulation and insulin resistance axis - marked by a decreased abundance of bacteria responsible for producing butyrate and acetate, coupled with increased glucose and HbA1c levels. The barrier function axis - involving a reduction in species that support gut barrier integrity and alterations in zonulin levels. These axes are not isolated but rather form a complex shift in microbiome composition alongside metabolic and regulatory processes. The described changes may not merely be markers of aging but could potentially be active contributors to age-related health decline.

The advantages of this work include the application of modern methods (neural network algorithms and XAI), the ability to obtain the contribution of individual predictors to the biological age calculation for a specific patient, the selection of a minimal necessary set of predictors (only 7 for the biochemical model and 45 species for the microbiota model), which facilitates their implementation in clinical practice, a wide age range (18-99 years), and high model performance metrics. However, the study also has limitations. It included only individuals of Caucasian population. Despite the minimal set of predictors, implementing the microbiota model in clinics might be challenging due to the requirement for sequencing biomaterial, which is not accessible to every healthcare institution.

In conclusion, this study developed two interpretable models for determining biological age using blood biochemical parameters and gut microbiota composition. Both models demonstrated high predictive accuracy (MAE ~ 6-7 years) and strong concordance with each other and with chronological age (correlation coefficient > 0.89). As the proposed models possess both global and local explainability, they hold future potential for application in monitoring the effectiveness of various interventions in clinical trials.

## Supplementary Material

Supplementary Table 1
